# Miscellaneous

**Published:** 1859-06

**Authors:** 


### Miscellaneous.

### A CALL.

The undersigned practitioners of Dentistry, believing that a National Association of Dentists, composed of delegates from State, county and local societies, and dental colleges, would be calculated to promote the best interests of the profession, respectfully suggest to the Dental Societies and Colleges throughout the country, the propriety of electing delegates to meet in convention at the Falls of Niagara, on the first Wednesday of August, 1859,—for the purpose of forming, if the assembled delegates shall deem it expedient, a National Association, upon a representative basis :

PENNSYLVANIA.

| | | | |
|---|---|---|---|
| J. De Haven White, | Philad'a. | J. Gilliams, | Philad'a. |
| Wm. W. Fouche, | do. | Jas. M. Harris, | do. |
| Daniel Neall, | do. | C. C. Williams, | do. |
| S. L. Mintzer, | do. | C. M. Pierce, | do. |
| J. H. McQuillen, | do. | T. L. Buckingham, | do. |
| J. Foster Flagg, | do. | Henry Townsend, | do. |
| Jas. E. Garretson, | do. | Charles Woodnnutt, | do. |
| Joseph R. Jefferies, | do. | S. Dillingham, | do. |
| C. A. Kingsbury, | do. | Charles E. Hopkins, | do. |
| George T. Barker, | do. | Andrew J. Watson, | do. |
| Louis Jack, | do. | Wm. Calvert, | do. |
| D. B. Whipple, | do. | A. R. Johnson, | do. |
| Wm. Gorges, | do. | J. J. Griffith, | do. |
| D. H. Goodwillie, | do. | G. M. Barstow, | do. |
| David Roberts, | do. | Spencer Roberts, | do. |
| Josiah Price, | W. Chester. | J. L. Snesserott, | Chambersb. |
| Jesse C. Green, | do. | Wm. F. Front, | do. |
| E. P. Worrall, | do. | C. S. Beck, | Wilkesb're. |
| C. M. Valentine. | do. | S. Atlee Bockins, | Columbia. |

OHIO.

| | | | |
|---|---|---|---|
| Charles Bonsall, | Cincinnati. | B. Strickland, | Cleveland. |
| James Taylor; | do. | W. H. Atkinson, | do. |
| H. R. Smith, | do. | B. F. Robinson, | do. |
| J. Taft, | do. | B. A. Holliwell, | do. |
| B. D. Wheeler, | do. | W. P. Horton, | do. |
| J. G. Cammeron, | do. | T. S. Slosson, | do. |
| J. Richardson, | do. | Charles R. Butler, | do. |
| C. P. Bailey, | Cuy. Falls. | J. F. Siddall, | Oberlin. |
| C. Palmer, | Warren. | S. P. Huntington, | Painesv'le. |
| J. C. Burroughs, | do. | E. J. Waye, | Sandusky. |
| B. F. Spellman, | Ravenna. | | |

MICHIGAN.

| | | | |
|---|---|---|---|
| C. F. Knowlton, | Detroit. | Wm. Cohoan, | Detroit. |
| J. A. White, | do. | J. H. Farmer, | do. |
| H. Benedict, | do. | G. L. Field, | do. |
| S. C. Whiting, | do. | | |

INDIANA.

| | | | |
|---|---|---|---|
| John F. Johnston, | Indianap's. | G. C. North, | Indianap's. |
| P. G. C. Hunt, | do. | T. M. Nichols, | do. |

ILLINOIS.

| | | | |
|---|---|---|---|
| E. A. Bogue, | Chicago. | L. P. Haskell, | Chicago. |
| T. P. Abell, | do. | Frank B. Ferris, | do. |
| Jas. Ketchum, | do. | P. W. Ferris, | do. |
| E. Honsinger, | do. | W. W. Allport, | do. |

MISSOURI.

| | | | |
|---|---|---|---|
| H. E. Peebles, | St. Louis. | Henry Barron, | St. Louis. |
| A. Blake, | do. | Isiah Forbes, | do. |
| W. A. Jones, | do. | C. W. Spaulding, | do. |
| D. L. Levett, | do. | Charles Hanover, | do. |

MISSISSIPPI.

J. H. Andrews.

### MISSISSIPPI VALLEY ASSOCIATION.

At the solicitation of some of the members of the society, I hereby call a meeting of the Mississippi Valley Association of Dental Surgeons, to take place on Wednesday, the 22d day of June, at 10 o'clock A. M., at the lecture-room of the Ohio College of Dental Surgery, to take into consideration the propriety of electing delegates to meet those to be appointed by other societies, to confer as to the expediency of forming a National Association, upon a representative basis, to be composed of delegates from the local societies and colleges, in accordance with the action of the Pennsylvania Association of Dental Surgeons; and other similar societies.

G. W. Keely,
*Oxford*, May 38, 1859. *President.*

### OHIO DENTAL COLLEGE ASSOCIATION.

Having been requested by the several members, I hereby call a meeting of the above Association to meet at the College, at 3 o'clock P. M. of Wednesday the 22d day of June, to take into consideration the propriety of electing delegates to meet those to be appointed by other societies, to confer as to the expediency of forming a National Association, upon a representative basis, to be composed of delegates from local societies and colleges, in accordance with the action of the Pennsylvania Association of Surgeon Dentists, and other similar societies. Charles Bonsall,
*Cincinnati*, May 26, 1859. *President.*

### CONSUMPTION OF GOLD.

The Medical and Surgical Reporter estimates that the five thousand practising Dentists in the United States consume, annnally, over two and a half millions of dollars' worth of gold, in plate and foil, for filling teeth.

